# Societal Perception of Animal Videos on Social Media—Funny Content or Animal Suffering? A Survey

**DOI:** 10.3390/ani14152234

**Published:** 2024-07-31

**Authors:** Alina Stumpf, Swetlana Herbrandt, Leia Betting, Nicole Kemper, Michaela Fels

**Affiliations:** 1 Institute for Animal Hygiene, Animal Welfare and Farm Animal Behaviour, University of Veterinary Medicine Hannover, Foundation, Bischofsholer Damm 15, 30173 Hannover, Germany; nicole.kemper@tiho-hannover.de (N.K.); michaela.fels@tiho-hannover.de (M.F.); 2 Statistical Consulting and Analysis, Center for Higher Education, TU Dortmund University, Vogelpothsweg 78, 44227 Dortmund, Germany; swetlana.herbrandt@tu-dortmund.de (S.H.); leia.betting@tu-dortmund.de (L.B.)

**Keywords:** animal videos, animal suffering, animal welfare, corporate social responsibility, social media, survey

## Abstract

**Simple Summary:**

This study aimed to find out how animal videos on social media are perceived by users. Numerous seemingly funny animal videos contain animal suffering. The results of a large-scale survey conducted in this study confirm that animal videos are very popular on social media as participants of all ages and education levels already watched them. However, it seems that viewers often do not realise when animals express signs of stress or discomfort in such videos. Only 45.8% of the participants often noticed animal suffering in videos, while 31% recognised animal well-being. Furthermore, participants of the survey stated that they would like to receive warning labels on videos showing animal suffering. The study highlights the need to inform social media users about animal welfare and provides the basis for developing strategies to reduce the spread of videos showing animal suffering on social media.

**Abstract:**

On social media, numerous animal videos are uploaded and viewed every day. However, these videos, which are apparently funny for humans, are often associated with animal suffering. In this study, 3246 participants of an online survey were asked about their personal perception of animal videos on social media, about recognising animal suffering in these videos, and about their respective reactions. A total of 98.5% of participants who used social media already saw animal videos. Participants most frequently viewed informative videos (52.9%), followed by funny/entertaining animal videos (41.8%). For 45.8% of participants, animal suffering was often recognisable in animal videos. Female participants were more likely to recognise animal suffering than male participants (*p* < 0.001), and participants living in a rural residence were more likely to recognise it than those from an urban residence (*p* = 0.017). Furthermore, 62.5% of participants had left a critical comment or disliked a video with animal suffering. Animal videos seem to be highly popular on social media, but animal suffering may go unnoticed in funny videos. The fact that 91.8% of participants want a warning label for animal suffering in videos shows that social media users would like to see animal welfare be given more prominence on social media.

## 1. Introduction

The usage of social media platforms is increasingly becoming a prominent factor in the way people engage in interpersonal communication. These platforms, including but not limited to Instagram, TikTok, and YouTube, have become an essential component of people’s daily lives, with many dedicating significant amounts of time to their usage. Social media is a very active and fast-moving domain [1]. In 2021, the platform Instagram counted 1.21 billion monthly active users worldwide [2].

Already in 2015, more than 400 hours of video content were uploaded to the platform YouTube every single minute [3]. Within this vast amount of content, animal videos make up a significant portion. On YouTube, videos featuring pets and animals generate an average of more than 6000 views per video, while those with “funny cats” in the title and description garner an average of 24,000 views [4]. Research has shown that watching cat videos on social media can increase positive emotions and decrease negative emotions among users [5]. Moreover, social media is often used as a procrastination strategy to avoid working on an unpleasant task, to change one’s mood, and for escapism [6]. Especially for young users, social media plays an important role in terms of their need to belong and their need for popularity [7,8].

Despite the widespread popularity of animal videos on social media platforms, little scientific research has been conducted to investigate the types of videos that are being viewed, including those that may depict animal suffering and raise concerns regarding animal welfare. In a scientific study, it was already confirmed that animal videos, which were categorised as funny and entertaining on different social media platforms, contained animal suffering that may not be recognised by laypeople, appearing harmless to them. This included the video contents “gloating”, “anthropomorphism”, and “challenges affecting animal welfare” [9]. In 2020, the Social Media Animal Cruelty Coalition (SMACC) was founded to draw attention to the problem of animal cruelty on social media. This was based on an extensive internet search that included more than 5000 animal videos with more than 5 billion views in total [10]. It is known that the use of animals for entertainment is often in connection to animal suffering and can lead to death [11].

Several platforms already prohibit the publication of content featuring animal cruelty in their guidelines. For instance, YouTube tightened the enforcement policies for staged animal rescue channels and videos in 2021 [12]. Instagram developed strategies to establish more animal welfare as well. Since 2017, some hashtags, such as #elephantselfie or #dolphinkiss, have been tagged with warning labels. But, unfortunately, this system is only a well-intended way to warn viewers of potential animal suffering content, but it has a lot of room for improvement and does not apply to hidden animal suffering in supposedly funny videos [13].

Given the vast number of clicks generated by animal videos, this topic still remains understudied. To date, there is limited research on how people perceive the welfare of animals in videos on social media, and existing studies on this subject are mainly dealing with the depiction of wild animals on social media [14,15]. There is currently a gap in knowledge about the social perception of the depiction of pets on social media.

Therefore, in this study, a large-scale survey was conducted with the aim of providing an overview of social media users’ experiences with pet videos, including a self-assessment of their ability to recognise animal suffering and welfare. Precise questions were used to determine the perception of animal videos and to identify the clues used to recognise animal suffering. Given that animal videos that are supposedly funny but may contain animal suffering are often very successful on social media, the hypothesis of this study was that these videos are very popular among users, but animal suffering is not always recognised. Therefore, it was asked how familiar the social media users are with certain “funny” videos showing animal suffering and how they obtain to these videos. Additionally, it was explored whether social media users are aware of the presence of animal suffering in videos, to what extent they trust themselves to recognise animal suffering, and what cues they use to make such judgments. Furthermore, it was determined how users deal with videos when they recognise animal suffering. A further hypothesis of this study was that there may be differences among demographic groups (e.g., age, gender, residence, etc.) in the way animal videos are judged and animal suffering is recognised. Based on the results, the popularity of critical animal videos in certain social media user groups should be identified, thereby creating the basis for targeted education about the risks of animal depictions on social media.

## 2. Materials and Methods

### 2.1. Survey Design

The scientific questionnaire was created using the programme LimeSurvey (V.3.23.1 + 200825, LimeSurvey GmbH, Hamburg, Germany). In the welcome text, the participants were briefed that the data of the survey would be analysed for research purposes and all answers would be handled anonymously.

The questionnaire consisted of 30 questions in the German language, which could be answered by ticking, mostly with a five-point Likert scale (never/almost never, rarely, sometimes, often, very often). There were also questions with binary yes/no response options and some with slider functions to specify the level of agreement in percent. The questionnaire was set up in a way that participants were directed toward the appropriate questions based on their previous answer. As a result, some participants might have answered fewer questions than others, depending on their answers to previous questions. Additionally, almost every question offered the option “no answer”. The survey was pretested with a group of 40 people who were not involved in the study design. The structure of the survey is shown in Table 1 and a copy of the original survey given to participants is included in the Supplementary Materials (Table S1).

The survey intentionally did not provide definitions of the terms “animal suffering” and “animal well-being” in order to be able to determine which behaviours the participants used to recognise them. However, specific behaviours were described in the survey (e.g., animals did not flee), and the participants of the survey were asked how often they saw these behaviours/animal reactions that may indicate poor or good animal welfare (Table S1 in Supplementary Materials).

### 2.2. Questionnaire Distribution

The survey was made accessible online between 26 July 2022 and 2 January 2023. It was aimed at all age groups and was spread via various channels, such as radio, newspapers, magazines, social media, and websites, to address a broad mass of society in German-speaking countries. A short link was generated leading to the survey, as well as a QR code that could be easily scanned with a smartphone.

The link to the survey was printed in various trade magazines and was published several times on popular online media. In addition, we announced the survey on personal social media accounts, as well as on the official university Instagram and Facebook accounts. Furthermore, the survey was distributed via mailing lists, among others, to universities, schools, and animal welfare organisations.

We also designed appealing flyers (500 pieces) with the QR code leading to the survey, which were displayed in schools, universities, sports clubs, medical surgeries, veterinary surgeries, pet-related shops, and in several public places around Hanover city (Germany). All participants were encouraged to distribute the survey in their circle of acquaintances and publish the link on their social media accounts as well as their websites.

### 2.3. Statistical Analysis

Data were downloaded from LimeSurvey (V.3.23.1 + 200825, LimeSurvey GmbH, Hamburg, Germany) and exported to Microsoft Excel (V.2301, Microsoft Corporation, Redmond, WA, USA). Statistical analysis was performed by use of the software R 4.2.2 [16].

At first, descriptive statistics were used to present the results of all analysed parameters. Thereby, the frequency distributions (percentages) of different responses to a question were calculated when a Likert scale was used in the questionnaire. Means and standard deviations were calculated when a continuous scale for agreement (0% to 100%) to a given statement was used in the questionnaire. Afterwards, statistical models were calculated for the following items: the frequency of viewing different types of animal videos, recognition of animal suffering/animal well-being in videos, and self-confidence in reliably recognising animal suffering. The level of significance was set at *p* < 0.05.

For the statistical models, only social media users were considered. Furthermore, the categories on the highest level of education were summarised to obtain sufficiently large groups (1: university degree, 2: apprenticeship, 3: none or school). Regarding group 3 (none or school), participants could originally select from various school-leaving qualifications as follows: Abitur (A-level), secondary school certificate, and basic school-leaving certificate. Additionally, options for “still in education” and “no qualification” were available, which were called “none”. Due to their small numbers, these responses were combined for statistical purposes. Thus, the group “none or school” encompasses all participants who have finished school, as well as those participants who were currently in education or have not attained a school-leaving qualification. Age categories were used to analyse the effect of age on the studied parameters (10, 25], (25, 30], (30, 40], (40, 50], (50, 60], (60, 90]). Due to the small number of participants who indicated their gender as diverse, these 20 questionnaires had to be excluded from the statistical models, and only ‘male’ and ‘female’ were used as genders in the models.

A mixed ordinal regression model [17] was applied to analyse the frequency of viewing different types of animal videos using the R package ordinal [18]. The individual participant was considered as a random effect, while the type of video, gender, age group, level of education, residence, and professional contact with animals as well as their interactions were set as fixed effects in the model. The model aimed to estimate the odds of selecting the next higher category on the Likert scale, with response options ranging from ‘1 = never/almost never’ to ‘5 = very often’. Pairwise comparisons for the fixed effects considered in the model were carried out using the Wald test by applying the R package emmeans [19]. Resulting *p*-values were adjusted using the Bonferroni method.

An ordinal regression model [17] was used to analyse the frequency of recognition of animal suffering and animal well-being in videos by using the R package ordinal [18]. Gender, age group, level of education, residence, and professional contact with animals as well as the interaction between the gender and age group were set as fixed effects in the model.

A linear regression model [17] was applied to analyse the self-confidence in reliably recognising animal suffering in videos, which was measured as a continuous variable (0% to 100%). For this purpose, the software R [16] was used. Gender, age group, level of education, residence, and professional contact with animals as well as the interaction between the gender and age group were set as fixed effects in the model.

## 3. Results

### 3.1. The Sample

In total, 4018 questionnaires were received. Since incomplete questionnaires were excluded from the study, a final sample of 3246 fully completed questionnaires was analysed.

In the following presentation of the results, the total number of participants per question (n_total_) may vary because, depending on the answer to a question, different follow-up questions may have been asked. Additionally, the option “no answer” could have been selected for almost every question.

### 3.2. Characteristics of the Participants

**Figure 1:**
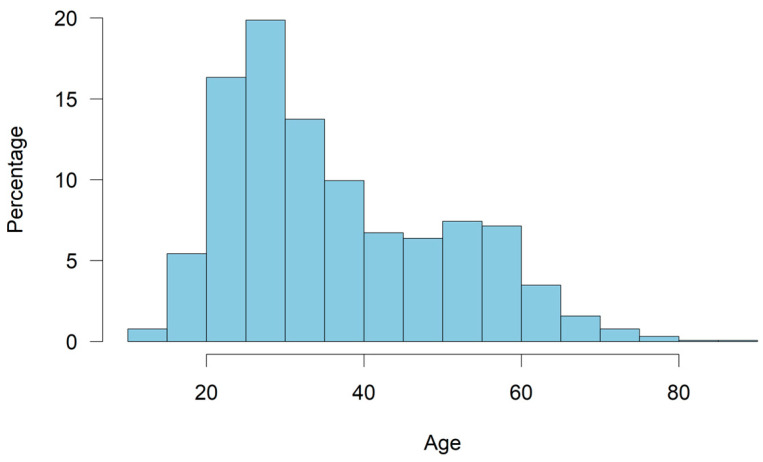
Percentage of different age groups of participants (n_total_ = 3246).

The distribution of age groups among participants is shown in Figure 1 (n_total_ = 3246). Participants were between 10 years and 88 years old. Most participants were aged between 25 and 30 years (19.9%), followed by those between 20 and 25 years (16.3%) and those between 30 and 35 years (13.7%). Participants were mainly female (86.5%) (n_total_ = 3195).

Almost half of the participants (46.3%) had a university degree, followed by a completed professional education (23.4%), school degree (28.3%), and those still in education (2.0%) (n_total_ = 3181). Contact with animals in their current or aspired profession was selected by 43.5% of the participants (n_total_ = 3172). A proportion of 47.0% lived in a rural residence, while 53.0% lived in an urban environment (n_total_ = 3190).

A total of 97.2% of participants own or have ever owned a pet (n_total_ = 3230). The most common pets were dogs (74.6%), followed by cats (55.5%), small pets, e.g., guinea pig, rabbit, chinchilla, hamster (55.3%), birds/fish (32.2%), exotics like reptiles and amphibians (9.5%), horses (19.5%), farm animals (8.4%), and others (2.3%).

### 3.3. Contact with Animal Videos on Social Media

**Figure 2:**
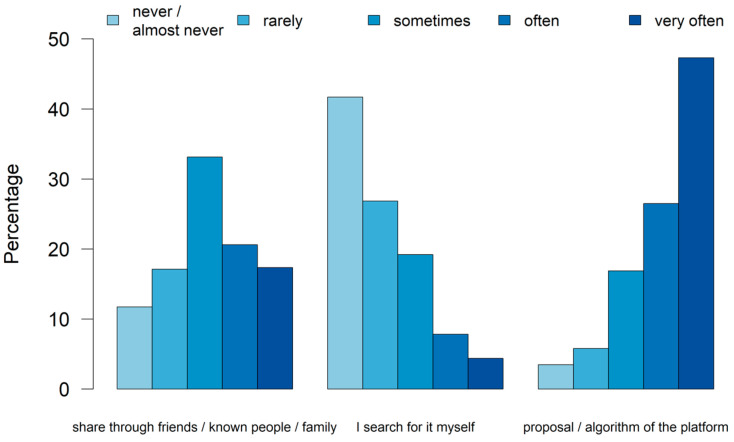
Percentage of how often participants viewed animal videos on social media platforms via sharing, searching, or proposal by the platform’s algorithm (n_total_ = 2982, 2983, 2977).

The majority (93.4%) of participants stated that they use social media platforms (n_total_ = 3246). Almost all of the participants who used social media platforms (98.5%) already viewed animal videos on these platforms (n_total_ = 3033). The most common way participants obtained animal videos was through the platform’s algorithm, followed by being shared by other users. Very few participants searched for animal videos themselves (Figure 2).

**Figure 3:**
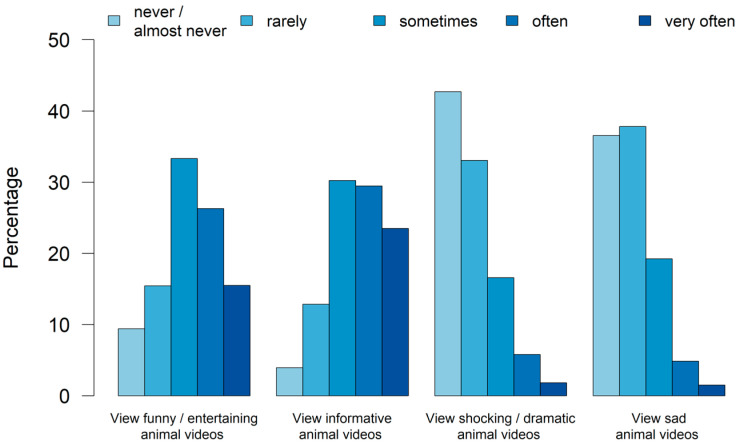
Percentage of how often participants viewed different types of animal videos (n_total_ = 2948, 2948, 2983, 2987).

The most frequently viewed animal videos were informative ones, while funny/entertaining ones ranked second (Figure 3). A total of 41.8% viewed funny/entertaining animal videos often/very often, while informative videos were seen often/very often by 52.9% of participants (n_total_ = 2984).

The results of the statistical model revealed that the frequency of watching different types of animal videos on social media was affected by different factors (Table 2 and Table S2). Pairwise comparisons (Table 3) showed that participants who had contact with animals in their current or aspired profession were more likely to view informative animal videos than persons who had no professional contact with animals (*p* < 0.001). In contrast, participants who had no contact with animals in their current or aspired profession were more likely to view funny/entertaining animal videos than those who had contact (*p* < 0.001). Participants with a university degree were also more likely to view funny/entertaining animal videos on social media than persons without university degrees or with school degrees (*p* < 0.001). Furthermore, participants living in an urban residence were more likely to view funny/entertaining animal videos than persons living in a rural residence (*p* = 0.004).

**Figure 4:**
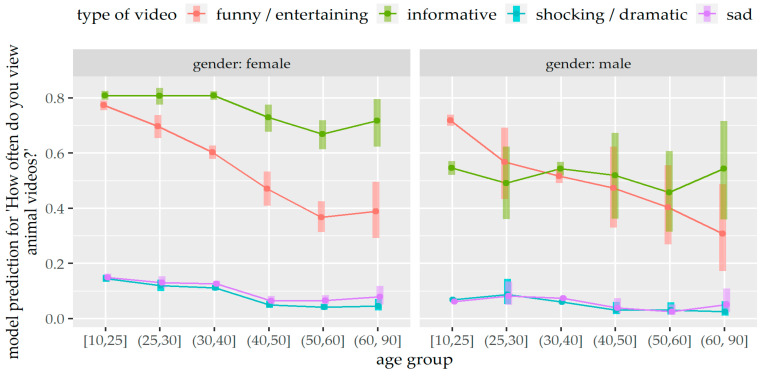
Prediction of the mixed ordinal regression model for watching different types of animal videos depending on gender and age group.

Additionally, there was an effect of the interaction between gender and age group on watching different types of animal videos (*p* < 0.001) (Table 2 and Figure 4). Female participants of all age groups were more likely to view informative videos than funny/entertaining videos (age group 10–25 years: *p* = 0.001, other age groups: *p* < 0.001), while there was no significant difference between informative and funny/entertaining videos for male participants of almost all age groups (*p* > 0.05), except for younger males between 10 and 25 years who were more likely to view funny/entertaining videos than informative ones (*p* < 0.001) (Figure 4 and Table S3). Furthermore, female participants of all age groups, except the age group 60–90 years (*p* > 0.05), were more likely to view informative videos than male participants (age groups 10–25, 25–30, 30–40 years: *p* < 0.001, age group 50–60 years: *p* = 0.008) (Figure 4 and Table S3).

### 3.4. Popular Animal Videos on Social Media

All specifically described video contents were already seen by more than 50% of participants (n_total_ = 2988); exact percentages are shown in Table 4. More than 90% already saw “animal mishaps” and “animal did something extraordinary”. The video content “animals wear human clothes or costumes” was seen by almost 85%.

The frequency of participants’ felt emotions while watching those described videos is also shown in Table 4. The emotion “anger/fright” was felt most (highest percentage on very often/often) while watching videos where an animal has been trapped and least (highest percentage on never/almost never) when watching videos where an animal performed something extraordinary. On the other hand, the emotion “fun/amusement” was felt most while watching videos where an animal performed something extraordinary and least while watching videos where an animal has been trapped.

### 3.5. Recognition of Animal Suffering

The analysis of questionnaires revealed that for 45.8% of the participants, animal suffering was often/very often recognisable in videos, for 35.3%, this was sometimes, for 14.8%, this was rarely, and only for 4.1%, animal suffering was never/almost never recognisable in those animal videos (n_total_ = 2977).

As shown in Table 5, female participants were more likely to recognise animal suffering in videos than male participants (*p* < 0.001), and participants who had contact with animals in their profession were more likely to notice animal suffering than those without this contact (*p* < 0.001). Participants with an apprenticeship/no degree/school degree were also more likely to recognise animal suffering in videos than those with a university degree (*p* < 0.001). Furthermore, participants living in a rural residence were more likely to recognise animal suffering in videos than those living in an urban residence (*p* = 0.017). The factors age group and the interaction between gender and age group did not affect the recognition of animal suffering in animal videos (*p* > 0.05).

**Figure 5:**
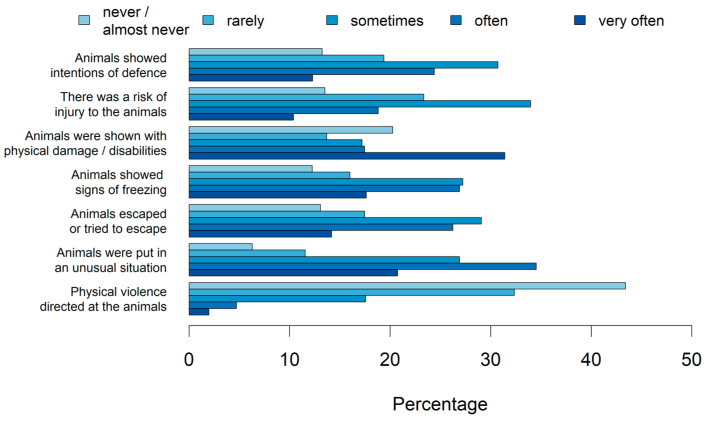
Percentage of different video contents revealing animal suffering to the participants (n_total_ = 2932, 2930, 2952, 2956, 2955, 2960, 2933).

Figure 5 shows which video content the participants saw that revealed animal suffering to them. Participants saw animal suffering-related content most (highest percentage on very often/often) when animals were put in an unusual situation and least (highest percentage on never/almost never) when there was physical violence directed to the animals.

### 3.6. Self-Confidence in Reliably Recognising Animal Suffering in Videos

The mean participants’ self-confidence in reliably recognising animal suffering in videos was 70.4 ± 23.8% relating to a scale from 0% (no self-confidence) to 100% (full self-confidence) (n_total_ = 3190).

The results of the statistical model are shown in Table 6. Female participants had a higher self-confidence in reliably recognising animal suffering in videos than male participants (*p* < 0.001), as well as participants who have contact with animals in their profession than those without this contact (*p* < 0.001). Participants living in a rural residence had a higher self-confidence in reliably recognising animal suffering in videos than those living in an urban residence (*p* = 0.016). Furthermore, participants with an apprenticeship/no degree/school degree had a higher self-confidence in reliably recognising animal suffering in videos than those with a university degree (*p* < 0.001). The factors age group and the interaction between gender and age group did not affect the self-confidence in reliably recognise animal suffering in videos (*p* > 0.05).

### 3.7. Recognition of Animal Well-Being

For 30.5% of participants, animal well-being was often/very often recognisable in animal videos, for 37.7%, this was sometimes, and for 25.5%, this was rarely (n_total_ = 2954). For 6.3% of participants, animal well-being was never/almost never recognisable in the videos (n_total_ = 2954).

The results of the statistical model revealed that male participants were more likely to recognise animal well-being in videos than female participants (*p* < 0.001), as well as participants who had no contact with animals in their profession than those with this contact (*p* = 0.007, Table 7). Furthermore, participants living in an urban residence were more likely to recognise animal well-being in videos than those living in a rural residence (*p* = 0.012). Participants with a university degree were also more likely to recognise animal well-being in videos than those with an apprenticeship (*p* = 0.002) (Table 7).

**Figure 6:**
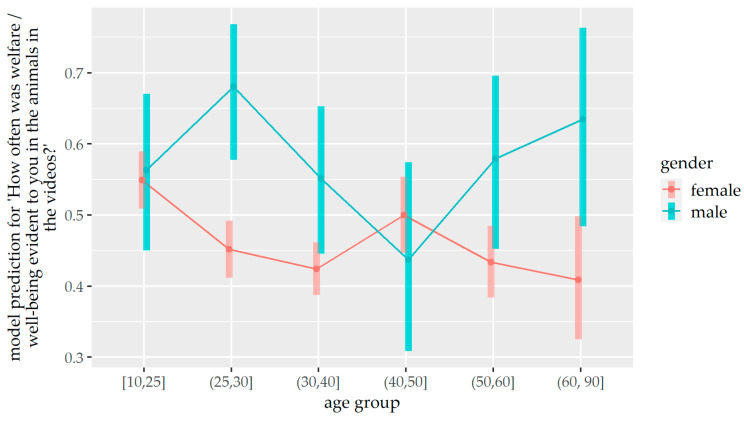
Prediction of the ordinal regression model for the recognition of animal well-being depending on gender and age group.

Additionally, there was an effect of the interaction between gender and age group on the recognition of animal well-being (*p* = 0.002) (Table 7 and Figure 6). Male participants of most age groups were more likely to recognise animal well-being in videos than female participants (25–30 years: *p* < 0.001, 30–40 years: *p* = 0.024, 50–60 years: *p* = 0.035, 60–90: *p* = 0.011). There was no significant difference between male and female of age groups 10–25 years and 40–50 years (*p* > 0.05).

**Figure 7:**
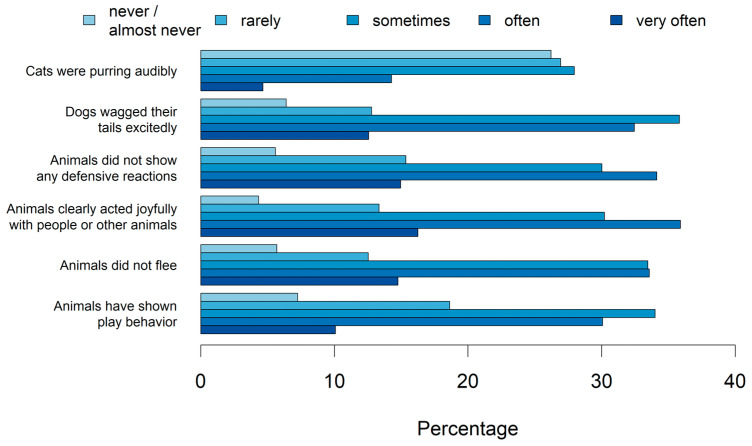
Percentage of different video contents revealing animal well-being to the participants (n_total_ = 2515, 2722, 2849, 2937, 2825, 2927).

Figure 7 shows which video content the participants saw that revealed animal well-being to them. Participants saw animal well-being most (highest percentage on very often/often) when animals clearly acted joyfully with people or other animals and least (highest percentage on never/almost never) when cats were purring audibly.

### 3.8. Personal Experiences

Slightly more than half of the participants (54.3%) had previously dealt with the topic of “animal welfare on social media” (n_total_ = 2947), and a total of 62.5% of the participants had ever given a critical comment or dislike to a video with animal suffering (n_total_ = 2915). Almost exactly half of the participants (50.7%) had ever reported a video with animal suffering to the platform (n_total_ = 2918). A total of 91.8% of participants wish to receive a warning notice for videos containing animal suffering on social media platforms in the future (n_total_ = 2814).

The mean level of agreement that animal welfare on social media should be given more importance was 88.9 ± 19.0% (n_total_ = 3207) relating to a scale from 0% (no agreement) to 100% (full agreement).

## 4. Discussion

The present study offers a range of insights into the experiences of users with animal videos on social media. The results support the hypothesis that animal videos are widely prevalent [20,21], as nearly all participants who used social media reported having seen animal videos there. The participants in our study were predominantly female, and there may be several reasons for this. Firstly, women generally spend more time on social media than men, and secondly, there are gender-specific differences in motivations for using social media as women are more interested in social topics [22,23]. Additionally, women seem to feel more engaged and willing to participate in surveys related to animals, as other survey studies have already shown [24,25]. Due to the large sample size (n_male_ = 411; n_female_ = 2764), it was nevertheless meaningful to analyse the influence of gender on the results of our study.

Notably, the way participants obtain animal videos is more often through random encounters, driven by the platforms’ algorithms, rather than actively searching for such content. It is already known that the algorithms of platforms are determined by what people primarily view, like, and share since the algorithm’s task is to prioritise what is most relevant to the user while filtering out what the user deems uninteresting [26]. This means that the animal videos suggested by the algorithm are derived from the user’s personal behaviour on social media.

The five video contents described in the survey were categorised as fun and entertainment on social media, but they offered plenty of animal suffering content, as our own research indicated. As expected, most of the participants were familiar with all these video contents. Particularly, videos featuring costumed animals or famous challenges generate many likes and positive comments on social media, a fact that was already observed with other videos featuring animal suffering which is not obviously animal cruelty [9,20].

Definitions of animal welfare or suffering have evolved over time. The five domains model highlights the absence of negative states, like pain, hunger, thirst, and fear, and emphasises addressing both physical and psychological dimensions, including behaviours such as anger, helplessness, and anxiety. But today, it is well-known that it is not enough to merely eliminate adverse conditions, and that animals should also experience positive emotional states [27] and have a “life worth living” [28]. This includes living and being kept in conditions appropriate to their species, especially for pets under human care who rely on being treated properly [10]. While the person who dresses up a dog in a tight costume may love this animal very much, the welfare of the animal may be negatively affected. Therefore, the present study aimed to investigate how familiar certain types of videos (that often contain animal suffering) are among social media users and whether users are aware of animal suffering in videos, i.e., what emotions they feel when watching the videos and how they deal with them.

An earlier study described that pet-related content on social media is consumed for joy because it offers a form of resistance and escape to the followers [29]; this is probably the explanation as to why these posts are so popular. In contrast, our participants frequently indicated that watching these videos evoked emotions of anger/fright. Possible reasons for this could be that nearly half of the respondents had professional contact with animals and may already be aware of the animal welfare issues depicted in the described videos. Another explanation could be that the participants may have answered based on social desirability bias. In this case, respondents tend to exaggerate things that make them look good and downplay the opposite [30,31].

Since the introductory text of the survey already indicated that it was a questionnaire from veterinarians, it is conceivable that the respondents felt uncomfortable admitting that they found these videos funny. Nevertheless, more than half of the participants have experienced enjoyment while watching these videos, with a particularly high proportion for challenges and animal mishaps, which very often involve animal suffering [9]. This may be reinforced by the humorous music frequently added to animal videos, which can manipulate the viewers’ mood, giving critical content a positive touch [32]. Of course, it is not known from the survey which videos the users saw, and perhaps not all of them contained animal suffering. Nevertheless, the survey asked about types of videos that are highly likely to contain animal suffering. Additionally, it is a common perspective that nearly everything related to animals is in some way funny, or at least tends to be humorous, which is why animal videos containing problematic content may be more likely to be overlooked when compared to human videos containing problematic content like racism [33]. If these animal videos are further titled with phrases like “try not to laugh” or labelled with hashtags such as “funny video,” it enables the viewer to immediately recognise the intention of the video. All in all, this confirms our hypothesis that animal suffering in videos is often not recognised and is instead classified as humorous.

Overall, participants reported watching informative animal videos most frequently, closely followed by funny animal videos. This result was statistically significant for female participants in every age group, while it was not significant for most age groups of male participants. One plausible explanation may be that women who are more interested in animals and their protection [34] also seek more information about animals, while men may be more inclined to watch entertaining videos. Furthermore, persons without professional contact with animals, persons from urban areas, and persons with a university degree consumed significantly more funny animal videos than informative ones. It is noticeable that persons with a more distant connection to animals, who likely have less knowledge about the animals’ needs and body language, tend to lean towards funny animal videos. Interestingly, these persons also noticed animal well-being in videos rather than animal suffering. Persons experiencing higher levels of stress in their professional careers [35] may also prefer funny animal videos, possibly as a means of stress reduction [5,6].

The hypothesis of this study that there may be differences between demographic groups in judging animal videos was also confirmed. Female participants stated that they recognised animal suffering more frequently and expressed more self-confidence in reliably identifying animal suffering than male participants. This could be attributed to the fact that women generally hold a more sceptical attitude towards animal well-being than men [34] and are more attentive to the emotions of animals than men [36]. This is also supported by the fact that, in our study, male participants more often recognised well-being in animal videos than female participants. An earlier study showed that women, unlike men, developed a greater sensitivity towards animal treatment when witnessing animal cruelty [37]. In our study, women demonstrated a higher propensity for recognising animal suffering, despite their greater consumption of informative videos, which typically have a lower likelihood of containing animal suffering. A more in-depth study would be necessary to better understand which concrete scenes with animal suffering the participants saw. Persons from rural areas and persons with professional contact to animals recognised animal suffering more frequently and expressed more self-confidence in reliably identifying animal suffering than the respective opposite. An explanation could be that people living closer to animals and working with them generally may have better knowledge of the animals’ needs and behaviour.

Interestingly, persons with a university degree reported seeing less animal suffering in the videos they watched, although they preferred funny animal videos. One could speculate that persons with a university degree are likely to engage in a higher consumption of funny animal videos as a coping mechanism for stress. This may lead to a reduced level of critical analysis regarding animal suffering in videos. Additionally, they expressed less self-confidence in reliably identifying animal suffering compared to the other participants. It can be assumed that persons with a higher level of education and extensive knowledge are also aware of the limits of their knowledge, including that concerning animal suffering.

The responses to the question of how animal suffering was recognised varied. Most participants were aware that animals with physical damage/disabilities may suffer. However, this leaves open the question of why agony breedings are still so popular [24], including on social media. It is not justifiable to cause physical or emotional harm to animals for human entertainment [11]. Agony breeding characteristics are breeding-related features that can cause pain, suffering, or harm to the animals. For instance, brachycephalic breeds (round face shape, short nose, large eyes) like the Pug or the French Bulldog have a predisposition for various health issues such as brachycephalic obstructive airway syndrome (BOAS) [38,39,40]. It is likely that not every physical damage is recognised as such since these are often not obvious to laypeople in the case of breeding-related defects.

Similarly, the responses regarding how participants recognised animal well-being varied, with answers such as “tail wagging,” “play behaviour,” and “not fleeing” being frequently chosen. However, these behaviours can also be stress responses [41,42]. Furthermore, an animal that does not flee or show any defensive reactions is not a measure of well-being but of the absence of animal suffering [43]. Despite nearly all participants had experience with owning pets, the results indicate that pet ownership alone is not sufficient to accurately interpret the behaviour of a particular species, and that fearful behaviour may be interpreted as relaxed [44]. This result is consistent with other studies that already found that owners frequently misunderstand their pets’ behaviour [45,46,47,48]. It is evident that subtle signs of stress in dogs, like appeasement gestures, are often overlooked or misinterpreted [48]. Similarly, cat owners frequently fail to recognise important indicators of stress in their pets [45].

Overall, we observed that participants associated animal well-being with indicators that can also be stress signals.

Approximately half of the participants have already engaged with the topic of animal welfare on social media, which leaves room for improvement. More than 50% have either disliked or left negative comments on videos featuring animal suffering. While this may be well-intentioned, it unfortunately has the opposite effect. Each interaction “feeds” the algorithm of the platform and ensures that the post becomes even more popular, while also leading to the continued recommendation of such content to the same person [26]. It is important to report these videos to the platform instead. This was completed by about half of the participants of the survey, which raises the question as to why content with animal suffering is not reported more often. It is encouraging to note that over 90% of participants desire a warning label for videos depicting animal suffering. Instagram already developed warnings for hashtags that are often associated with animal suffering [13], showing that such a system can be implemented. In summary, there is a clear need for increased attention to the topic of animal welfare on social media, and most respondents showed a willingness to address this issue.

## 5. Conclusions

The present study confirmed that animal videos are very popular on social media, as participants of all age groups and different education levels indicated that they knew of them. However, it can be assumed that social media users do not always reliably recognise whether animal suffering is present in the videos. This is particularly indicated by the fact that a warning label for videos depicting animal suffering would be highly appreciated by the participants of this survey. Such a warning system for animal welfare-related content may prevent the normalisation of animal suffering on social media by raising user awareness. User awareness, in turn, can prevent such video content from becoming successful and being imitated, thus causing even more animal suffering. Therefore, a warning label may promote animal welfare and simultaneously protect those who prefer not to view such content. Thus, warning labels could have a useful educational effect, allowing users to quickly skip the posts containing animal suffering.

The results of this survey show how important it is to inform social media users about animal welfare-related topics. It can provide the basis for developing strategies to reduce the dissemination of videos with animal suffering by raising the awareness of this topic. This study significantly advances contemporary animal welfare standards.

## Figures and Tables

**Table 1:** Structure of the survey.

**Section 1**	Eligibility for participation	By indicating the year of birth; for underage participants (<18 years), parental consent was required
**Section 2**	General information	Questions about owning pets and one’s personal usage of different social media platforms (time spent on different platforms per day) *
**Section 3**	Contact with animal videos on social media	Determining if participants have already seen animal videos on social media (yes/no) Detecting how participants came across animal videos (own search, sharing, suggestion of the platform) Detecting the frequency (1: never/almost never, 2: rarely, 3: sometimes, 4: often, 5: very often) of viewing different types of animal videos (1: funny/entertaining, 2: informative, 3: shocking/dramatic, 4: sad)
**Section 4**	Perception of animal videos on social media	Five different video types were described, and participants were asked if they had ever seen them * (1: animal performed something extraordinary such as facial expressions/gestures/vocalisations, 2: challenge for owner and pet, 3: animal has been trapped, 4: animal mishaps, 5: animals wore human clothes or costumes/not medically necessary clothing or protection from the weather). The description was not related to a specific video, but to general video content that is often found on social media. Because of the copyright, no actual animal videos were shown in the survey. Detecting which kind of feelings such videos evoke (1: anger/fright, 2: fun/amusement) in general
**Section 5**	Recognition of animal suffering and animal well-being in videos	Participants were asked whether and how they recognise animal suffering (seven described situations) and animal well-being (six described situations) in videos * Furthermore, participants were asked to estimate their self-confidence in accurately recognising animal suffering in videos (percent) *
**Section 6**	Personal experience	Questions about the importance of animal welfare on social media * Detecting how participants deal with animal videos (like, share, comment, report on platform) *
**Section 7**	Personal data	Gender, educational background, contact with animals in current or aspired profession, living environment (urban/rural)

* For detailed information about the individual questions of the survey, please see the Supplementary Materials (Table S1).

**Table 2:** Results of the mixed ordinal regression model for watching different types of animal videos on social media platforms.

Model Term	df1	df2	F-Ratio	*p*-Value
type of video	3	∞	885.799	<0.001
gender	1	∞	56.917	<0.001
age group	5	∞	17,817.875	<0.001
residence	1	∞	0.173	0.677
level of education	2	∞	2593	0.075
contact with animals in the current or aspired profession	1	∞	19,128.002	<0.001
type of video/gender	3	∞	12,592	<0.001
type of video/age group	15	∞	165,963.880	<0.001
type of video/residence	3	∞	6.626	<0.001
type of video/level of education	6	∞	13.123	<0.001
type of video/contact with animals in the current or aspired profession	3	∞	146,650.318	<0.001
gender/age group	5	∞	3581.206	<0.001
type of video/gender/age group	15	∞	25,233.012	<0.001

**Table 3:** Results of pairwise comparisons of different factors influencing the watching of different types of videos.

Type of Video	Compared Groups	Odds Ratio	SE	z Ratio	*p* Value	2.5%	97.5%
	Contact with Animals in the Current or Aspired Profession						
funny/entertaining	contact/no contact	0.917	<0.001	−108.476	<0.001	0.916	0.919
informative	contact/no contact	1.241	0.001	190.974	<0.001	1.238	1.244
	residence						
funny/entertaining	urban/rural	1.244	0.095	2.859	0.004	1.071	1.446
informative	urban/rural	0.847	0.065	−2.162	0.031	0.729	0.985
	level of education						
funny/entertaining	apprenticeship/none or school	1.270	0.128	2.379	0.052	0.998	1.617
funny/entertaining	university/none or school	1.602	0.139	5.418	<0.001	1.301	1.972
funny/entertaining	university/apprenticeship	1.261	0.135	2.170	0.090	0.976	1.627
informative	apprenticeship/none or school	1.111	0.111	1.051	0.879	0.874	1.413
informative	university/none or school	0.830	0.072	−2.145	0.096	0.674	1.022
informative	university/apprenticeship	0.747	0.079	−2.742	0.018	0.579	0.964

**Table 4:** Popularity of specifically described video contents and the frequency of the emotions “anger/fright” and “fun/amusement” while watching those video contents in percent.

Video Content	Percentage of Participants Who Were Aware of This Content (n_total_ = 2988)	Percentages of the Emotion “Anger/Fright”	Percentages of the Emotion “Fun/Amusement”
Animal has been trapped	51.4%	45.8% very often 24.2% often 16.9% sometimes 7.4% rarely 5.7% never/almost never (n_total_ = 1530)	0.6% very often 4.0% often 11.7% sometimes 19.7% rarely 64.0% never/almost never (n_total_ = 1534)
Challenge that owners set with an animal	78.9%	27.0% very often 25.3% often 25.0% sometimes 13.6% rarely 9.1% never/almost never (n_total_ = 2344)	2.1% very often 9.4% often 24.7% sometimes 26.5% rarely 37.3% never/almost never (n_total_ = 2351)
Animal wore human clothes or costumes	84.3%	34.1% very often 23.4% often 23.4% sometimes 11.4% rarely 7.7% never/almost never (n_total_ = 2505)	1.0% very often 4.9% often 14.9% sometimes 22.7% rarely 56.5% never/almost never (n_total_ = 2508)
Animal mishaps	91.1%	22.7% very often 21.0% often 29.9% sometimes 17.2% rarely 9.2% never/almost never (n_total_ = 2703)	4.6% very often 15.5% often 30.2% sometimes 23.2% rarely 26.5% never/almost never (n_total_ = 2715)
Animal did something extraordinary (facial expressions, gestures, vocalisations)	96.4%	18.3% very often 23.0% often 27.1% sometimes 17.1% rarely 14.5% never/almost never (n_total_ = 2863)	6.2% very often 19.0% often 33.7% sometimes 22.7% rarely 18.4% never/almost never (n_total_ = 2872)

**Table 5:** Results of the ordinal regression model for recognition of animal suffering in videos on social media platforms (n_total_ = 2793).

	Estimate	Odds Ratio	Std. Error	df1	df2	F-Ratio	*p*-Value
never/almost never/rarely	−3.322	0.036	0.137				
rarely/sometimes	−1.532	0.216	0.108				
sometimes/often	0.226	1.253	0.103				
often/very often	1.802	6.061	0.110				
gender = male	−1.114	0.328	0.242				
age group = (25, 30]	0.329	1.389	0.116				
age group = (30, 40]	0.492	1.636	0.113				
age group = (40, 50]	0.023	1.023	0.136				
age group = (50, 60]	0.159	1.173	0.133				
age group = (60, 90]	0.339	1.404	0.204				
residence = urban	−0.167	0.846	0.070				
level of education = apprenticeship	0.034	1.035	0.100				
level of education = university	−0.451	0.637	0.090				
Do you have contact with animals in your current or aspired profession? = yes	0.479	1.614	0.071				
gender = male/age group = (25, 30]	−0.049	0.952	0.334				
gender = male/age group = (30, 40]	−0.269	0.764	0.332				
gender = male/age group = (40, 50]	−0.186	0.831	0.370				
gender = male/age group = (50, 60]	0.059	1.061	0.365				
gender = male/age group = (60, 90]	−0.077	0.926	0.455				
Model Term							
gender				1	∞	106.490	<0.001
age group				5	∞	1.832	0.103
residence				1	∞	5.686	0.017
level of education				2	∞	20.564	<0.001
Do you have contact with animals in your current or aspired profession?				1	∞	45.124	<0.001
gender/age group				5	∞	0.240	0.945

**Table 6:** Results of the linear regression model for self-confidence in reliably recognising animal suffering in videos (n_total_ = 2762).

	Estimate	Std. Error	df1	df2	F-Ratio	*p*-Value
(Intercept)	66.080	1.265				
gender = male	−7.774	3.078				
age group = (25, 30]	4.009	1.447				
age group = (30, 40]	4.855	1.411				
age group = (40, 50]	4.469	1.694				
age group = (50, 60]	5.301	1.668				
age group = (60, 90]	9.587	2.553				
residence = urban	−2.135	0.885				
level of education = apprenticeship	−0.014	1.249				
level of education = university	−4.145	1.117				
Do you have contact with animals in your	10.650	0.894				
current or aspired profession? = yes						
gender = male/age group = (25, 30]	−0.487	4.282				
gender = male/age group = (30, 40]	−4.951	4.216				
gender = male/age group = (40, 50]	−8.802	4.740				
gender = male/age group = (50, 60]	−1.141	4.665				
gender = male/age group = (60, 90]	−8.627	5.635				
Model Term						
gender			1	2746	67.054	<0.001
age group			5	2746	1.619	0.151
residence			1	2746	5.818	0.016
level of education			2	2746	10.170	<0.001
Do you have contact with animals in your current or aspired profession?			1	2746	142.037	<0.001
gender/age group			5	2746	1.270	0.274

**Table 7:** Results of the ordinal regression model for the recognition of animal well-being in videos on social media platforms (n_total_ = 2776).

	Estimate	Odds Ratio	Std. Error	df1	df2	F-Ratio	*p*-Value
never/almost never/rarely	−2.952	0.052	0.125				
rarely/sometimes	−0.995	0.370	0.104				
sometimes/often	0.636	1.889	0.103				
often/very often	2.669	14.419	0.125				
gender = male	0.057	1.059	0.242				
age group = (25,30]	−0.392	0.676	0.115				
age group = (30,40]	−0.504	0.604	0.113				
age group = (40,50]	−0.197	0.821	0.137				
age group = (50,60]	−0.465	0.628	0.133				
age group = (60, 90]	−0.567	0.567	0.200				
residence = urban	0.177	1.193	0.070				
level of education = apprenticeship	−0.114	0.892	0.099				
level of education = university	0.193	1.212	0.089				
Do you have contact with animals in your current or aspired profession? = yes	−0.193	0.825	0.071				
gender = male/age group = (25, 30]	0.893	2.443	0.339				
gender = male/age group = (30, 40]	0.455	1.577	0.332				
gender = male/age group = (40, 50]	−0.312	0.732	0.385				
gender = male/age group = (50, 60]	0.529	1.697	0.369				
gender = male/age group = (60, 90]	0.866	2.377	0.437				
Model Term							
gender				1	∞	16.386	<0.001
age group				5	∞	1.498	0.187
residence				1	∞	6.275	0.012
level of education				2	∞	6.496	0.002
Do you have contact with animals in your current or aspired profession?				1	∞	7.342	0.007
gender/age group				5	∞	2.914	0.012

## Data Availability

The data that support the findings of this study are available from the corresponding author upon reasonable request.

## Supplementary Materials

The following supporting information can be downloaded at https://www.mdpi.com/article/10.3390/ani14152234/s1, Table S1: Copy of the original survey given to participants; Table S2: Model adjustment of the mixed ordinal regression model for watching different types of animal videos on social media platforms; Table S3: Results of the pairwise comparisons of the interaction between gender and age group on watching different types of animal videos.
