# Supplementary material for: Societal Perception of Animal Videos on Social Media—Funny Content or Animal Suffering? A Survey
Source: Animals (Basel). 2024 Jul 31;14(15):2234. doi: 10.3390/ani14152234 (PMC11310948; doi:10.3390/ani14152234)
Supplement: Supplementary file 1 [file animals-14-02234-s001.zip › Supplementary Materials_S1_S2_S3.pdf]

Supplementary Materials

Table S1. Copy of the original survey given to participants.

Question Number	Question	Answer options	Question type	Follow up question
**Q1**	Please enter your year of birth.		Number	<2004: Q2<br>2004 or more: Q3
**Q2**	For participants under 18 years of age, the consent of a parent or guardian to participate in this survey is required.	1. The condition is fulfilled<br>2. The condition is not fulfilled	Single choice	1: Q3<br>2: End of the survey
**Q3**	Do you own or have you ever owned a pet?	1. Yes<br>2. No<br>3. No answer	Single choice	1: Q4<br>2: Q5<br>3: Q5
**Q4**	Which kind of pet(s)?	1. Dog(s)<br>2. Cat(s)<br>3. Small pets, e.g. guinea pig, bunny, chinchilla, hamster<br>4. Exotic(s), e.g. reptile, amphibian<br>5. Bird(s) or fish<br>6. Horse(s)<br>7. Farm animal(s)<br>8. Other	Multiple choice	
**Q5**	Do you use social media platforms?	1. Yes<br>2. No	Single choice	1: Q6<br>2: Q26

**Q6**	Please estimate your daily using time in minutes per day for each platform.<br>1. Facebook<br>2. Instagram<br>3. Snapchat<br>4. TikTok<br>5. YouTube	Range between 0 and 240 minutes per day per platform	Number	
**Q7**	Have you had any contact to animal videos on social media platforms?	1: Yes<br>2: No	Single choice	1: Q8<br>2: Q22
**Q8**	How did you get to animal videos on social media?<br>1. Share through friends/known people/family<br>2. I search for myself<br>3. Proposal/algorithm of the platform	1: Never/almost never<br>2: Rarely<br>3: Sometimes<br>4: Often<br>5: Very often	Single choice	
**Q9.1**	How often do you watch animal videos of following types on social media platforms?<br>1. Funny/entertainment<br>2. Informative<br>3. Shocking/dramatic<br>4. Sad	1: Never/almost never<br>2: Rarely<br>3: Sometimes<br>4: Often<br>5: Very often	Single choice	
**Q9.2**	How often do you share animal videos of following types on social media platforms?<br>1. Funny/entertainment<br>2. Informative<br>3. Shocking/dramatic<br>4. Sad	1: Never/almost never<br>2: Rarely<br>3: Sometimes<br>4: Often<br>5: Very often	Single choice	
**Q10**	Have you ever seen videos featuring an animal did something extraordinary (facial expressions/gestures/vocalisations)?	1: Yes<br>2: No	Single choice	1: Q11<br>2: Q12

**Q11**	What kind of emotions did you feel while watching?<br>1. Anger/fright<br>2. Fun/amusement	1: Never/almost never<br>2: Rarely<br>3: Sometimes<br>4: Often<br>5: Very often	Single choice	
**Q12**	Have you ever seen videos featuring a challenge (owner with pet), e.g. cat vs. Cucumber-Challenge, Bark at your dog-Challenge?	1: Yes<br>2: No	Single choice	1: Q13<br>2: Q14
**Q13**	What kind of emotions did you feel while watching?<br>1. Anger/fright<br>2. Fun/amusement	1: Never/almost never<br>2: Rarely<br>3: Sometimes<br>4: Often<br>5: Very often		
**Q14**	Have you ever seen videos featuring an animal trap, e.g. an animal was lured to run into a transparent wall?	1: Yes<br>2: No	Single choice	1: Q15<br>2: Q16
**Q15**	What kind of emotions did you feel while watching?<br>1. Anger/fright<br>2. Fun/amusement	1: Never/almost never<br>2: Rarely<br>3: Sometimes<br>4: Often<br>5: Very often	Single choice	
**Q16**	Have you ever seen funny videos featuring animal mishaps, e.g. a cat fell into a swimming pool, a hamster fell out of its hamster wheel?	1: Yes<br>2: No	Single choice	
**Q17**	What kind of emotions did you feel while watching?<br>1. Anger/fright<br>2. Fun/amusement	1: Never/almost never<br>2: Rarely<br>3: Sometimes<br>4: Often<br>5: Very often	Single choice	
**Q18**	Have you ever seen videos featuring animals wearing human clothes or costumes?	1: Yes<br>2: No	Single choice	

**Q19**	What kind of emotions did you feel while watching?<br>1. Anger/fright<br>2. Fun/amusement	1: Never/almost never<br>2: Rarely<br>3: Sometimes<br>4: Often<br>5: Very often	Single choice
**Q20**	How often did you recognise animal suffering in those videos?	1: Never/almost never<br>2: Rarely<br>3: Sometimes<br>4: Often<br>5: Very often	Single choice
**Q21**	How did you recognise animal suffering in videos?<br>1. Physical violence against the animal<br>2. The animal was put in an extraordinary situation, e.g. wearing human clothes<br>3. The animal flied or tried to flee<br>4. The animal froze<br>5. The animal had physical damage or exhibited agony breeding characteristics, e.g. "funny" body condition/face<br>6. There was a risk of injury to the animal<br>7. The animal defended itself	1: Never/almost never<br>2: Rarely<br>3: Sometimes<br>4: Often<br>5: Very often	Single choice
**Q22**	How often did you recognise animal well-being in those videos?	1: Never/almost never<br>2: Rarely<br>3: Sometimes<br>4: Often<br>5: Very often	Single choice

**Q23**	How did you recognise animal well-being in videos?<br>1. The animal showed play behaviour<br>2. The animal did not flee<br>3. The animal was excited and interacted friendly with humans or other animals<br>4. The animal did not defend itself<br>5. The dog wagged its tail<br>6. The cat purred	1: Never/almost never<br>2: Rarely<br>3: Sometimes<br>4: Often<br>5: Very often	Single choice
**Q24**	Please answer "yes" or "no".<br>1. Have you ever given a critical comment or a dislike to a video featuring animal suffering?<br>2. Have you ever reported a video featuring animal suffering?	1: Yes<br>2: No<br>3: No answer	Single choice
**Q25**	Please indicate your level of agreement as a percentage.<br>1. The topic of "animal welfare on social media" should be given more importance<br>2. I trust myself to reliably recognise animal suffering in videos	0% to 100%<br>(100% = completely agreement)	Choose per cent
**Q26**	Please answer "yes" or "no".<br>1. Have you ever dealt with the topic "animal welfare on social media"?<br>2. Do you wish to receive a warning notice for videos containing animal suffering on social media?	1: Yes<br>2: No<br>2: No answer	Single choice
**Q27**	Please indicate the gender you feel you belong to.	1: Male<br>2: Female<br>3: Diverse<br>4: No answer	Single choice

**Q28**	Please indicate your highest level of education.	1: No degree<br>2: Still in education<br>3: Secondary school certificate<br>4: Intermediate school leaving certificate<br>5: A-level certificate<br>6: Apprenticeship<br>7: University degree<br>8: No answer	Single choice
**Q29**	Do you have contact with animals in your current or aspired profession?	1: Yes<br>2: No<br>3: No answer	single choice
**Q30**	In what area is your current residence located?	1: Urban<br>2: Rural<br>3: No answer	Single choice

Table S2. Model adjustment of the mixed ordinal regression model for watching different types of animal videos on social media platforms.

	Estimate	Odds Ratio	Std. Error
never / almost never | rarely	-3770	0.023	0.041
rarely | sometimes	-1853	0.157	0.030
sometimes | often	0.001	1.001	7.443×10^{-4}
often | very often	1709	5522	7.443×10^{-4}
type of video = informative	0.525	1690	8.041×10^{-4}
type of video = shocking / dramatic	-2716	0.066	8.041×10^{-4}
type of video = sad	-2525	0.080	8.041×10^{-4}
gender = male	-0.289	0.749	7.958×10^{-4}
age group = (25,30]	-0.392	0.676	0.115
age group = (30,40]	-0.811	0.444	7.958×10^{-4}
age group = (40,50]	-1345	0.261	0.139
age group = (50,60]	-1772	0.170	0.134

age group = (60, 90]	-1680	0.186	0.226
residence = urban	0.219	1.244	0.076
level of education = apprenticeship	0.239	1.270	0.101
level of education = university	0.471	1.602	0.087
Do you have contact with animals in your current or aspired profession? = yes	-0.086	0.917	7.958×10^{-4}
gender = male:age group = (25,30]	-0.275	0.760	0.291
gender = male:age group = (30,40]	-0.063	0.939	7.958×10^{-4}
gender = male:age group = (40,50]	0.302	1.352	0.333
gender = male:age group = (50,60]	0.444	1.559	0.336
gender = male:age group = (60, 90]	-0.070	0.933	0.445
type of video = informative:gender = male	-0.975	0.377	8.041×10^{-4}
type of video = shocking / dramatic:gender = male	-0.549	0.578	8.041×10^{-4}
type of video = sad:gender = male	-0.684	0.504	8.041×10^{-4}
type of video = informative:age group = (25,30]	0.379	1.460	0.138
type of video = shocking / dramatic:age group = (25,30]	0.164	1.178	0.139
type of video = sad:age group = (25,30]	0.231	1.260	0.136
type of video = informative:age group = (30,40]	0.806	2.240	8.041×10^{-4}
type of video = shocking / dramatic:age group = (30,40]	0.507	1.660	8.041×10^{-4}
type of video = sad:age group = (30,40]	0.619	1.857	8.041×10^{-4}
type of video = informative:age group = (40,50]	0.887	2.428	0.165
type of video = shocking / dramatic:age group = (40,50]	0.166	1.181	0.174
type of video = sad:age group = (40,50]	0.409	1.505	0.169
type of video = informative:age group = (50,60]	1027	2.792	0.157
type of video = shocking / dramatic:age group = (50,60]	0.409	1.506	0.171
type of video = sad:age group = (50,60]	0.866	2.377	0.164
type of video = informative:age group = (60,90]	1165	3.207	0.266
type of video = shocking / dramatic:age group = (60, 90]	0.410	1.507	0.286
type of video = sad:age group = (60, 90]	0.960	2.612	0.276
type of video = informative:residence = urban	-0.385	0.681	0.092
type of video = shocking / dramatic:residence = urban	-0.277	0.758	0.094
type of video = sad:residence = urban	-0.302	0.739	0.092

type of video = informative:level of education = apprenticeship	-0.134	0.875	0.120
type of video = shocking / dramatic:level of education = apprenticeship	-0.290	0.748	0.123
type of video = sad:level of education = apprenticeship	-0.232	0.793	0.121
type of video = informative:level of education = university	-0.657	0.518	0.104
type of video = shocking / dramatic:level of education = university	-0.790	0.454	0.107
type of video = sad:level of education = university	-0.789	0.454	0.105
type of video = informative:Do you have contact with animals in your current or aspired profession? = yes	0.302	1.353	8.040×10^{-4}
type of video = shocking / dramatic:Do you have contact with animals in your current or aspired profession? = yes	0.429	1.536	8.040×10^{-4}
type of video = sad:Do you have contact with animals in your current or aspired profession? = yes	0.094	1.099	8.040×10^{-4}
type of video = informative:gender = male:age group = (25,30]	0.070	1.072	0.347
type of video = shocking / dramatic:gender = male:age group = (25,30]	0.763	2.146	0.356
type of video = sad:gender = male:age group = (25,30]	0.734	2.083	0.350
type of video = informative:gender = male:age group = (30,40]	0.059	1.061	8.041×10^{-4}
type of video = shocking / dramatic:gender = male:age group = (30,40]	0.221	1.248	8.041×10^{-4}
type of video = sad:gender = male:age group = (30,40]	0.438	1.550	8.041×10^{-4}
type of video = informative:gender = male:age group = (40,50]	0.049	1.050	0.408
type of video = shocking / dramatic:gender = male:age group = (40,50]	0.041	1.042	0.437
type of video = sad:gender = male:age group = (40,50]	0.143	1.154	0.424
type of video = informative:gender = male:age group = (50,60]	-0.053	0.948	0.396
type of video = shocking / dramatic:gender = male:age group = (50,60]	0.054	1.055	0.437
type of video = sad:gender = male:age group = (50,60]	-0.452	0.636	0.442
type of video = informative:gender = male:age group = (60, 90]	0.574	1.775	0.524

type of video = shocking / dramatic:gender = male:age group = (60, 90]	0.323	1.381	0.601
type of video = sad:gender = male:age group = (60, 90]	0.576	1.779	0.554

Table S3. Results of the pairwise comparisons of the interaction between gender and age group on watching different types of animal videos.

age group	gender	type of video	odds ratio	SE	z-ratio	p-value	2.5%	97.5%
[10,25]	female	informative / (funny / entertaining)	1.246	0.075	3.667	0.001	1.064	1.459
	male	informative / (funny / entertaining)	0.470	0.028	-12.607	< 0.001	0.401	0.550
(25,30]	female	informative / (funny / entertaining)	1.819	0.219	4.971	< 0.001	1.324	2.499
	male	informative / (funny / entertaining)	0.735	0.241	-0.938	1.000	0.310	1.745
(30,40]	female	informative / (funny / entertaining)	2.790	0.167	17.121	< 0.001	2.382	3.268
	male	informative / (funny / entertaining)	1.116	0.067	1.828	0.405	0.953	1.307
(40,50]	female	informative / (funny / entertaining)	3.024	0.458	7.302	< 0.001	2.028	4.511
	male	informative / (funny / entertaining)	1.197	0.454	0.475	1.000	0.440	3.258
(50,60]	female	informative / (funny / entertaining)	3.478	0.500	8.677	< 0.001	2.381	5.081
	male	informative / (funny / entertaining)	1.244	0.459	0.590	1.000	0.469	3.295
(60, 90]	female	informative / (funny / entertaining)	3.995	1.042	5.311	< 0.001	2.008	7.948
	male	informative / (funny / entertaining)	2.673	1.216	2.162	0.184	0.805	8.877

age group	type of video	gender	odds ratio	SE	z-ratio	p-value	2.5%	97.5%
[10,25]	funny / entertaining	male / female	0.749	5.963×10-4	-362.717	< 0.001	0.748	0.750
	informative	male / female	0.283	3.196×10-4	-1.117.328	< 0.001	0.282	0.283
[25,30]	funny / entertaining	male / female	0.569	0.166	-1.933	0.053	0.322	1.008
	informative	male / female	0.230	0.067	-5.053	< 0.001	0.130	0.407
[30,40]	funny / entertaining	male / female	0.703	7.225×10-4	-342.698	< 0.001	0.702	0.705
	informative	male / female	0.281	4.137×10-4	-862.427	< 0.001	0.280	0.282
[40,50]	funny / entertaining	male / female	1.013	0.337	0.040	0.968	0.528	1.945
	informative	male / female	0.401	0.141	-2.601	0.009	0.202	0.798
[50,60]	funny / entertaining	male / female	1.168	0.393	0.462	0.644	0.605	2.257
	informative	male / female	0.418	0.138	-2.636	0.008	0.218	0.799
[60, 90]	funny / entertaining	male / female	0.699	0.311	-0.805	0.421	0.292	1.671
	informative	male / female	0.468	0.206	-1.722	0.085	0.197	1.111
